# Clinical Pathway for the Diagnosis and Management of Patients With Relapsing–Remitting Multiple Sclerosis: A First Proposal for the Peruvian Population

**DOI:** 10.3389/fneur.2021.667398

**Published:** 2021-10-21

**Authors:** César Caparó-Zamalloa, Victor Velásquez-Rimachi, Nicanor Mori, Wenzel Ivan Dueñas-Pacheco, Andrely Huerta-Rosario, Chandel Farroñay-García, Roberto A. Molina, Carlos Alva-Díaz

**Affiliations:** ^1^Basic Research Center in Dementias and Central Nervous System Demyelinating Diseases, Instituto Nacional de Ciencias Neurológicas, Lima, Peru; ^2^Neurosonología, Clínica Delgado, Lima, Peru; ^3^Facultad de Medicina, Universidad Nacional Mayor de San Marcos, Lima, Peru; ^4^Red de Eficacia Clínica y Sanitaria (REDECS), Lima, Peru; ^5^Grupo de Investigación Neurociencia, Efectividad Clínica y Salud Pública, Universidad Científica del Sur, Lima, Peru; ^6^Servicio de Neurología, Departamento de Medicina y Oficina de Apoyo a la Docencia e Investigación (OADI), Hospital Daniel Alcides Carrión, Callao, Peru; ^7^Servicio de Neurología, Hospital Nacional María Auxiliadora, Lima, Peru; ^8^Facultad de Medicina Hipólito Unanue, Universidad Nacional Federico Villarreal, Lima, Peru; ^9^Instituto Nacional de Salud (INS), Lima, Peru

**Keywords:** multiple sclerosis, relapsing-remitting, patient care management, critical pathways, Peru

## Abstract

**Background:** Relapsing–remitting multiple sclerosis (RRMS) is a subtype of degenerative inflammatory demyelinating disease of multifactorial origin that affects the central nervous system and leads to multifocal neurological impairment.

**Objectives:** To develop a clinical pathway (CP) for the management of Peruvian patients with RRMS.

**Methods:** First, we performed a literature review using Medline, Embase, Cochrane, ProQuest, and Science direct. Then, we structured the information as an ordered and logical series of five topics in a defined timeline: (1) How should MS be diagnosed? (2) How should a relapse be treated? (3) How should a DMT be initiated? (4) How should each DMT be used? and (5) How should the patients be followed?

**Results:** The personnel involved in the care of patients with RRMS can use a series of flowcharts and diagrams that summarize the topics in paper or electronic format.

**Conclusions:** We propose the first CP for RRMS in Peru that shows the essential steps for diagnosing, treating, and monitoring RRMS patients based on an evidence-based medicine method and local expert opinions. This CP will allow directing relevant clinical actions to strengthen the multidisciplinary management of RRMS in Peru.

## Introduction

Multiple sclerosis (MS) is a degenerative inflammatory demyelinating disease of multifactorial origin that affects the central nervous system (CNS) and leads to multifocal neurological impairment. It occurs more frequently in young adults aged between 15 and 35, being more frequent in women (1). It is currently considered a complex disease influenced by genetic, epigenetic, and environmental factors (2).

The overall prevalence has been estimated at 30 per 100,000 inhabitants (3). The regions with the highest prevalence of MS are North America with 191.2 cases per 100,000 inhabitants, and Europe, with 96 to 200 cases per 100,000 inhabitants. On the other hand, Asia and sub-Saharan Africa have a lower prevalence with <0.22 per 100,000 inhabitants. In South America, a prevalence of 5.24 cases per 100,000 inhabitants was recorded, particularly in Panama and Argentina (Patagonia), with an estimated prevalence and incidence of 17.2/100,000 and 1.4/100,000, respectively (4, 5). In Peru, the prevalence calculated for Lima was 7.69 per 100,000 inhabitants (6). The current perception is that there is an increase in the prevalence and incidence of this disease that could be explained by increased disease awareness, better access to diagnostic tools, longer survival, and more sensitive diagnostic criteria resulting in better case detection (7, 8).

MS has a varied clinical presentation in which two recognized clinical phenotypes have been described and are characterized by their activity and progression: (1) relapsing MS and (2) progressive MS. However, a clinically isolated syndrome (CIS) and a radiologically isolated syndrome (RIS) have also been described and should be taken into account (9).

Clinical pathways (CP) are a helpful tool for continuous quality improvement in healthcare and facilitate the integration of clinical practice guidelines, protocols, and local algorithms. The advantages of CP are based on optimizing integrated mechanisms that include the appropriate activities necessary to manage specific medical problems (10, 11). Therefore, they allow standardizing diagnosis and treatment while always prioritizing common sense and clinical experience, positively influencing the best professional training, and facilitating teamwork (12, 13).

In 2019, the Peruvian Society of Neurology published a clinical guideline for managing patients with MS to provide neurologists with a valid, updated tool to treat these patients in a comprehensive manner (14). However, a more practical and user-friendly tool was needed to achieve greater acceptance among Peruvian neurologists, thus standardizing the management of Peruvian patients based on quality external information adapted to our context by a group of thematic experts.

Therefore, we developed a CP to diagnose and manage patients with relapsing–remitting MS (RRMS) with summary versions of the recommendations through evidence-based algorithms.

## Materials and Methods

We developed a CP route with the following methodological design criteria aimed at (1) developing a structured multidisciplinary care plan; (2) channeling the translation of guides or tests to local structures; (3) describing the steps of the therapeutic course using a route, an algorithm, a guide, a protocol, or another “inventory of actions”; and (4) standardizing care for a specific clinical problem, procedure, or episode of care in a specific population (15).

To achieve this, we recruited a group of Peruvian neurologists working at public hospitals with over 5 years of experience managing MS and a methodological team with experience in synthesizing evidence. Then, we conducted a review of the literature using different sources (Medline, Embase, Cochrane, ProQuest, and Science direct) with “Multiple Sclerosis, Relapsing–Remitting” as the MeSH term. We identified relevant evidence that covers issues related to the care of patients with RRMS, and during six meetings, we planned and designed five key topics developed in this CP: (1) diagnosis, (2) relapse treatment, (3) initiation of disease-modifying treatment (DMT), (4) use of each DMT, and (5) follow-up.

## Results

**Figure 1:**
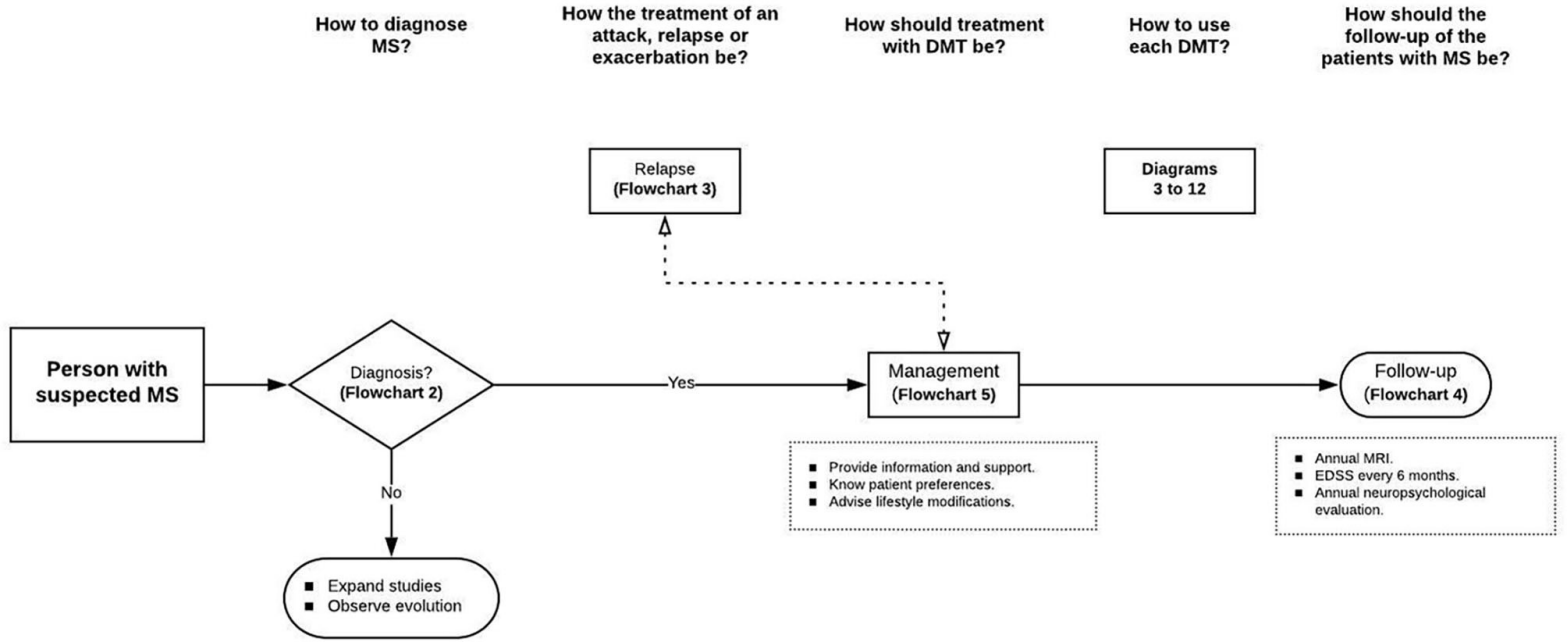
Clinical pathway for diagnosis and treatment of RRMS. Source: Authors.

We organized the present CP as an ordered and logical series of resolved topics in a consecutively defined timeline (Figure 1). In addition, we accompanied this CP with a series of flow diagrams that neurologists can use. This CP for the care of patients with MS could be used in paper or electronic format and consists of the following questions:

### How Should MS Be Diagnosed?

The initial evaluation must define whether the patient presents with a typical CIS (9) (Additional File 1). Patients with ≥2 lesions on magnetic resonance imaging (MRI) have a high probability of developing MS (16, 17). The McDonald 2017 criteria must be applied to confirm the diagnosis of MS. There are no specific considerations for the Peruvian population except ruling out tuberculosis (18) (Additional File 2). Finally, the prognosis should be assessed according to the following unfavorable outcome factors: age >40 years, male sex, African American or Latin American ethnicity, polyfocal presentation, involvement of the afferent system, and partial or no recovery, all of which can increase the risk of developing aggressive forms of MS (19).

### How Should a Relapse Be Treated?

**Figure 2:**
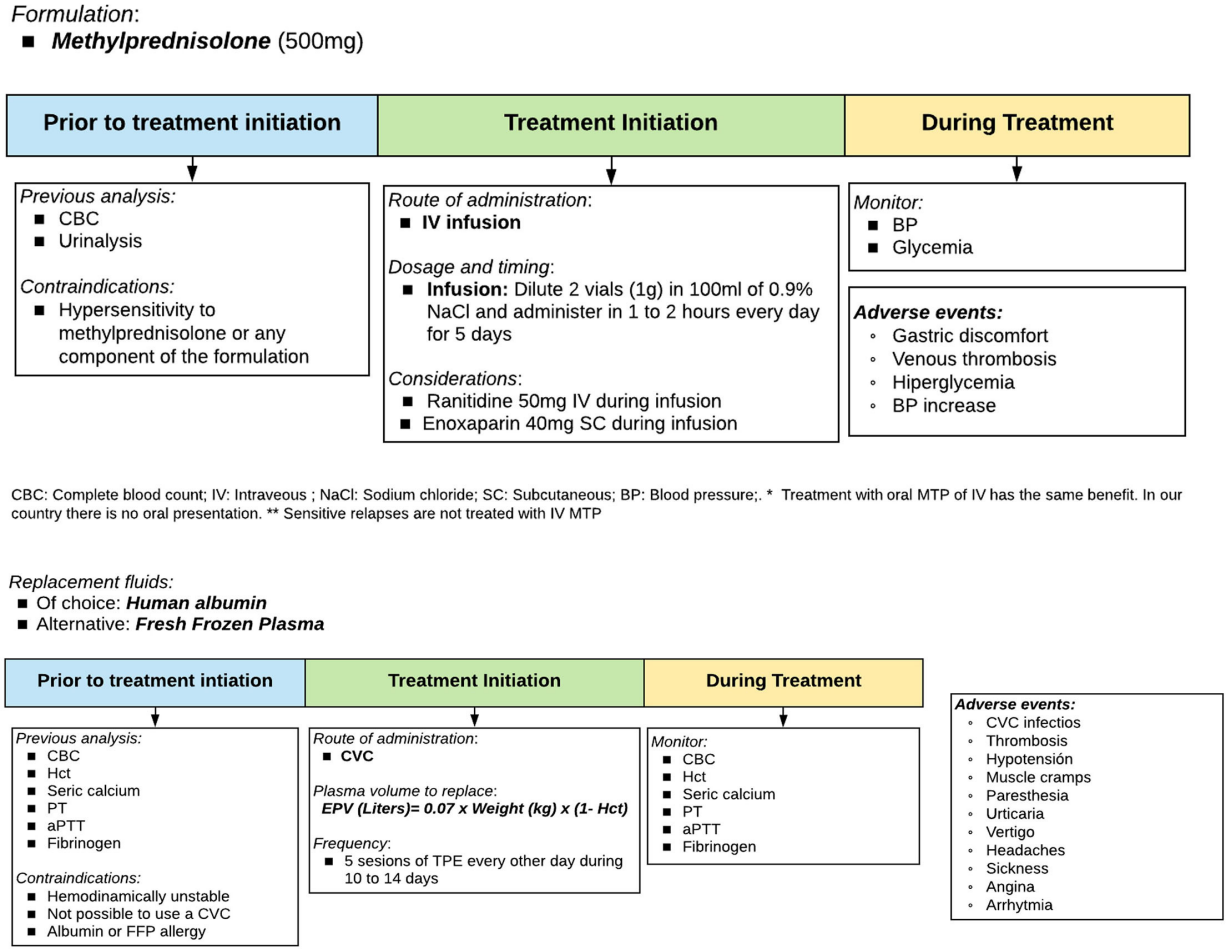
Therapeutic protocol for relapses: Methylprednisolone and therapeutic plasma exchange. Source: Authors.

**Figure 3:**
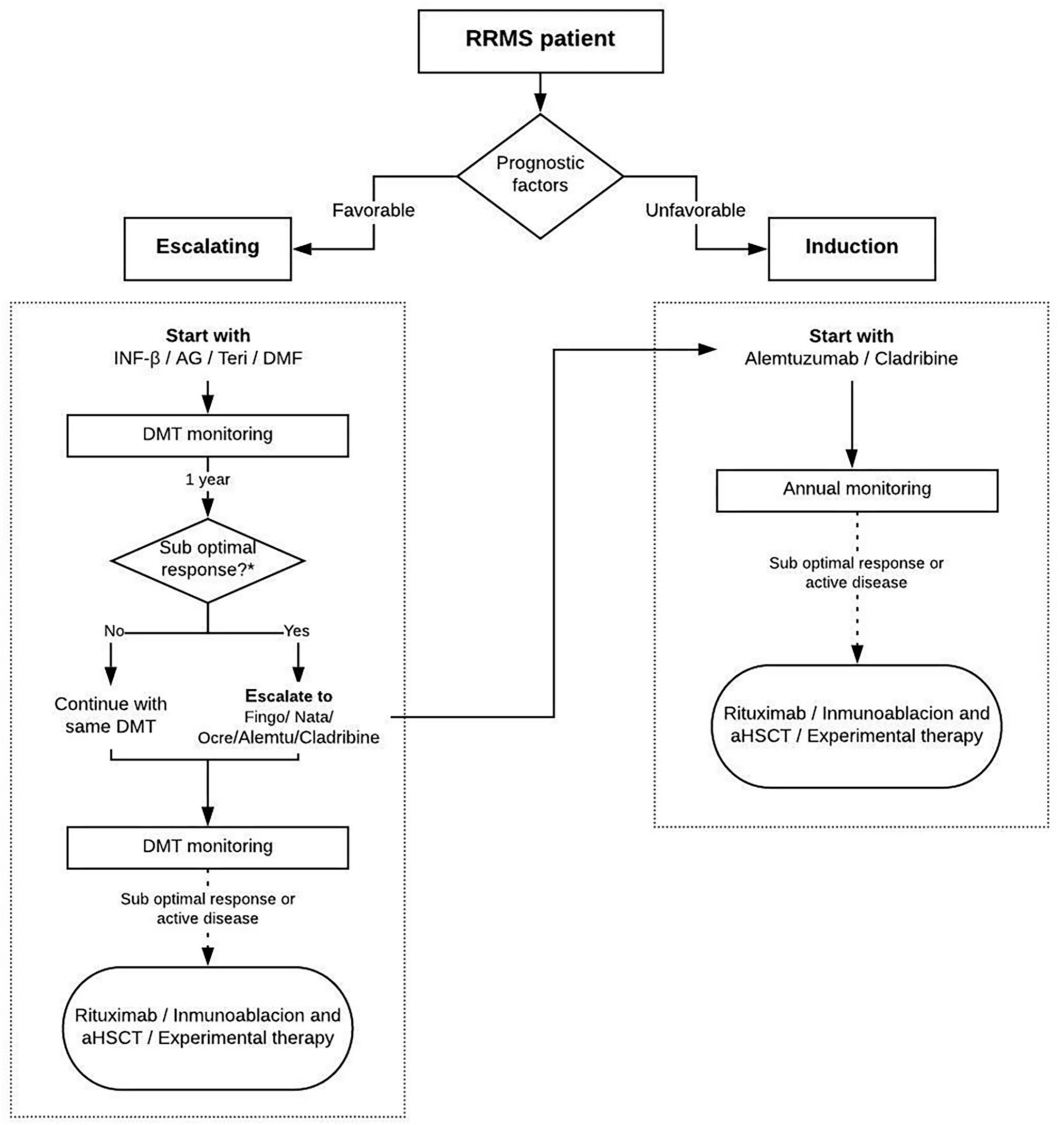
Treatment approach in RRMS. Source: Freedman et al. and Giovannoni et al. (27, 28).

In patients presenting moderate to severe relapse (20–22) (Additional File 3), the first option is intravenous high-dose methylprednisolone pulse therapy (6, 23, 24) (Figure 2). Alternatively, oral methylprednisolone could be used since evidence of a similar effect exists (25–28), but tablets >8 mg are not available in Peru. Another alternative could be oral prednisone 1,250 mg daily; however, 25 tablets per day of prednisone 50 mg make this alternative not suitable. If the patient does not respond favorably or cannot comply with methylprednisolone therapeutic protocol, treatment with therapeutic plasma exchange should be considered (23, 29) (Figure 3).

### How Should a DMT Be Initiated?

**Figure 4:**
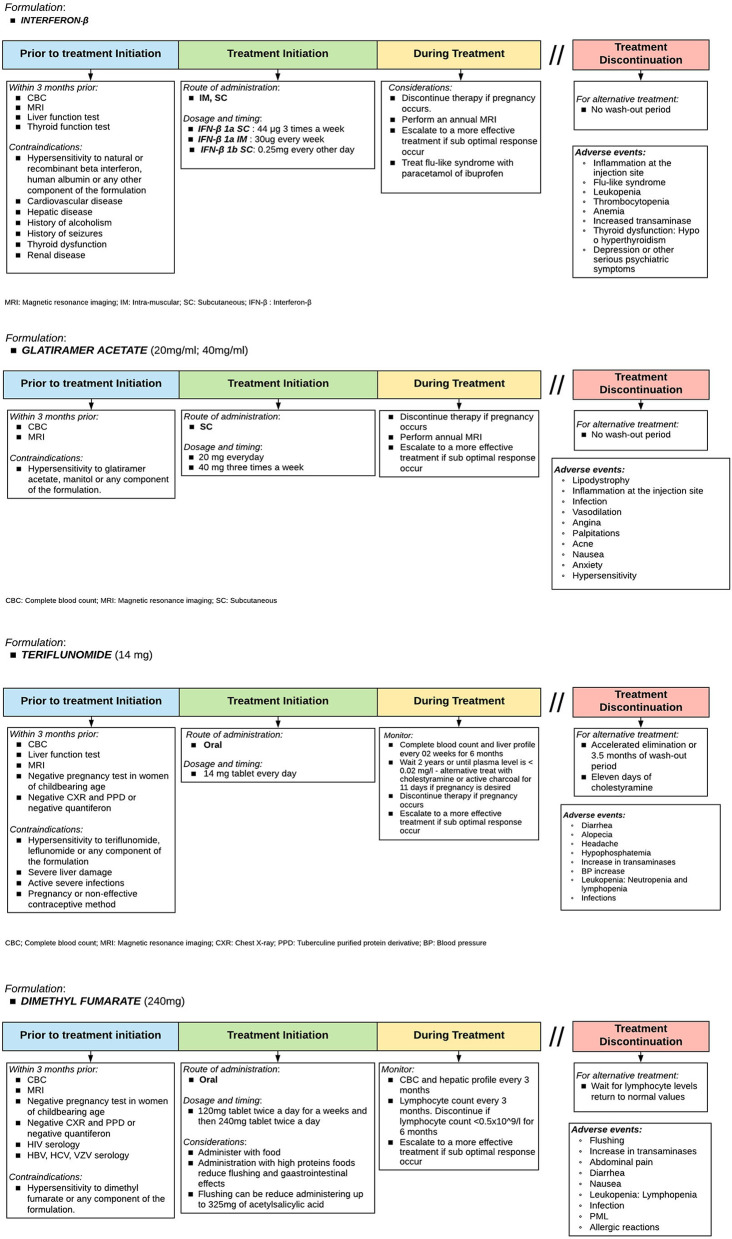
Therapeutic protocol for induction: Interferon-β, glatiramer acetate, teriflunomide, and dimethyl fumarate. Adapted from Sorensen et al. (33).

The choice of a DMT should be made according to the patients' characteristics, the evaluation of prognostic factors, the risk–benefit balance of the treatment options, and the experience of the treating neurologist (30–32) (Figure 4). It should be noted that this algorithm is only a reference since high efficacy can also be achieved with first-line therapies depending on the clinical factors. The Modified Rio score should be used to evaluate the treatment response with IFN, teriflunomide, and glatiramer acetate at 12 months later (33–35). For the remaining DMTs, clinical and imaging assessments should be performed every year (Additional File 4).

### How Should Each DMT Be Used?

**Figure 5:**
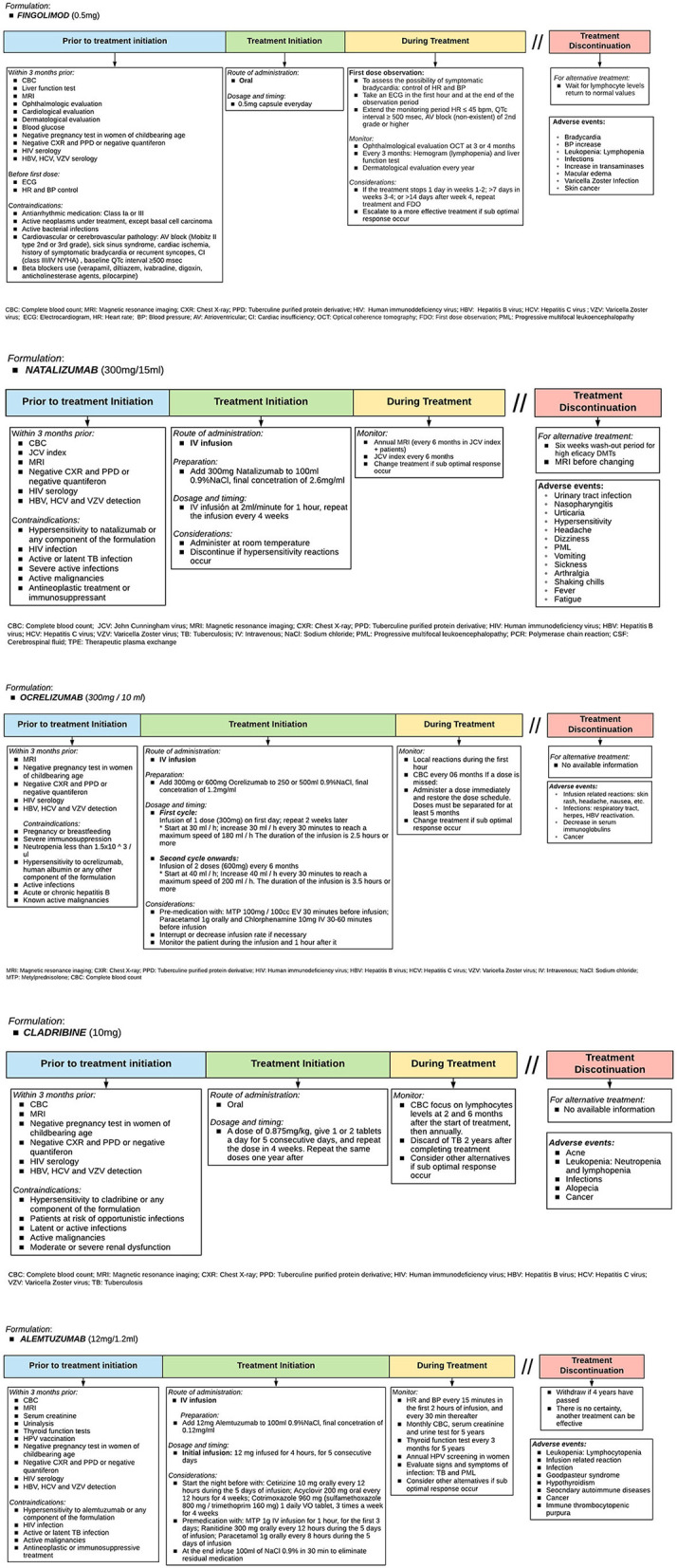
Therapeutic protocol for escalating: Fingolimod, natalizumab, ocrelizumab, cladribine, and alemtuzumab. Adapted from Sorensen et al. (33).

**Figure 6:**
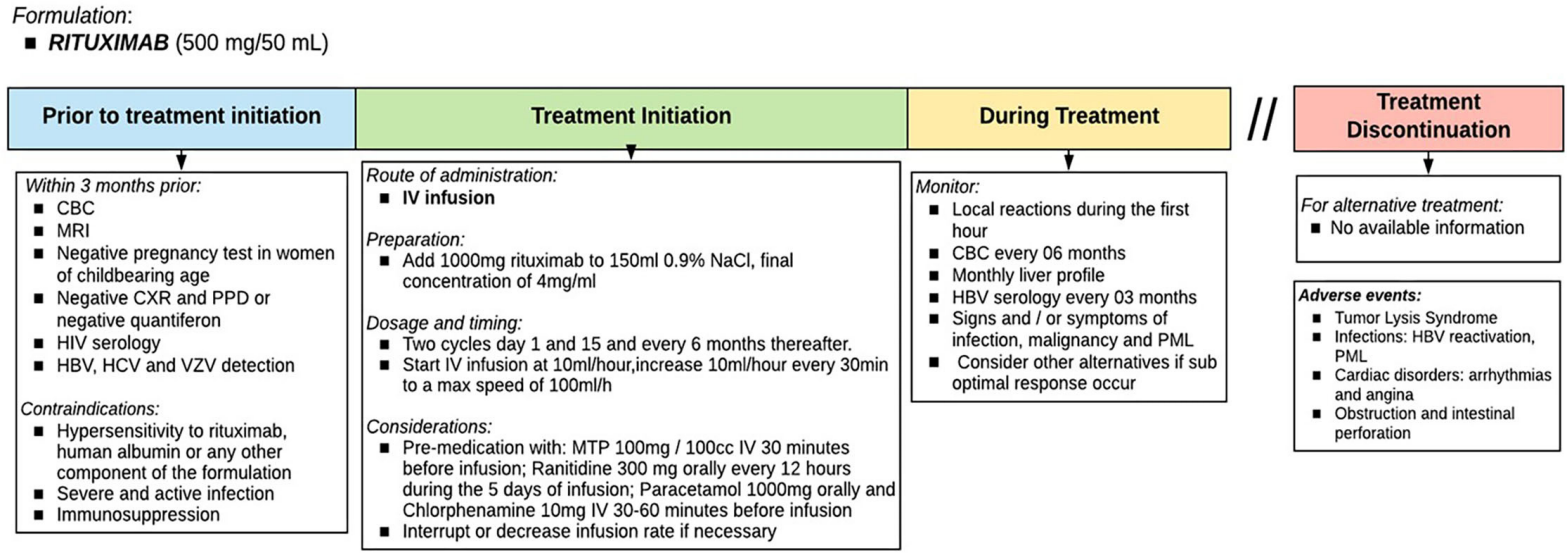
Therapeutic protocol for failure: Rituximab. The indication of rituximab as a DMT in RRMS has not yet been approved in Peru and is used as an off-label indication. Source: Authors.

There are several DMT for RRMS treatment (14, 36, 37). However, specific tests are needed before initiating treatment. It is also important to know patient preferences and provide advice on individual general recommendations (38). Figures 5, 6 show diagrams describing the use of each DMT (1, 38–50).

### How Should the Patients Be Followed?

After treatment initiation, a brain MRI must be obtained annually, and a spinal MRI should be requested only if spinal cord symptoms occurred (51). In addition, experts suggest a comprehensive clinical assessment including biannually Expanded Disability Status Scale (EDSS) and annually neuropsychological evaluations (Expert consensus) (33, 34).

No evidence of disease activity (NEDA) considering no relapses, no increase of disability (as measured with EDSS), and no new or active MRI lesions can also be used as a treatment objective (52, 53).

## Discussion

Patients with RRMS are young and present a chronic and disabling evolution, making it necessary to perform a multidisciplinary approach. This disease is characterized by an often-unpredictable course making diagnosis difficult and the choice of the adequate DMT for each patient challenging (1, 14, 54).

The clinical variability of RRMS requires a multidisciplinary intervention by healthcare professionals, making adequate resource management a necessity to reduce morbidity and disability, and thereby improve the quality of life of individuals with this disease. The use of the proposed CP will allow patients to receive relevant, timely clinical interventions and significantly reduce the use of hospital resources, without negatively affecting the length of stay and hospital costs (54–56).

Our CP indicates the steps to be followed in the initial phase of diagnosis, then in the treatment and monitoring phase, and finally during patient follow-up. We propose a current and adapted list of diagrams to guide DMT use in the Peruvian population based on the previous proposal by Sorensen et al. (38), which explains the tests to be made before, during, and after initiating treatment in chronological order as well as possible treatment schemes that neurologists can choose and how to perform monitoring.

There are limitations for MS diagnosis in Peru due to the difficult access to specialists access and MRI (16); therefore, there is a delay between the first clinical outbreak and the confirmatory diagnosis of up to 3.2 years (57). In addition, there are difficulties in accessing timely treatment because public institutions only have interferon and glatiramer acetate as the DMT scale. Moreover, to access induction DMT, it is necessary to evaluate a case for at least 12 months, classify it as a therapeutic failure and make a request for the new treatment that takes an average of 4–6 months, delaying the start of treatment with more significant disability and lower quality of life (16).

We organized several meetings with methodologists and neurologists to adapt the selected external information on the management of RRMS to the national context to resolve this. Our CP is innovative and is the first approach to integrating processes oriented at the diagnostic and therapeutic resources available for RRMS in Peru.

## Conclusions

We have proposed the first CP for RRMS in Peru with a chronological description of the steps to follow for the diagnosis, treatment, and follow-up of RRMS patients. This will be a helpful tool for Peruvian neurologists in order to carry out a systematic process for the care of persons with MS.

We hope that the use of this CP will have a real impact on continuous improvement in the care and quality of health provided by neurologists, which will be reflected by the satisfaction perceived by Peruvian RRMS patients. Finally, we believe that this CP for diagnosing and managing patients with RRMS will be an essential tool for encouraging correct and methodical approaches to the disease based on quality scientific-technical evidence, generating standard use of treatments and rational use of health resources.

## Author Contributions

CA-D, VV-R, NM, CC-Z, and WD-P participated in meetings to plan, design, and elaborate on the clinical pathway. VV-R, AH-R, RM, and CF-G identified relevant studies to support the clinical pathway. VV-R, AH-R, and CA-D participated in the writing of the manuscript. All authors designed the study and approved the final version of the manuscript.

## Funding

Hoffmann–La Roche Pharmaceutical provided financing for the execution and publication of the clinical pathway.

## Conflict of Interest

The authors declare that this study received funding from Hoffmann-La Roche Pharmaceutical. The funder had the following involvement in the study: provided financing for the execution and publication of the clinical pathway.

## Publisher's Note

All claims expressed in this article are solely those of the authors and do not necessarily represent those of their affiliated organizations, or those of the publisher, the editors and the reviewers. Any product that may be evaluated in this article, or claim that may be made by its manufacturer, is not guaranteed or endorsed by the publisher.

## Supplementary Material

Additional File 1 MS diagnostic approach. Source: Huamani et al. (16) and Kuhler et al. (17).

Additional File 2 Diagnostic protocol: Mc Donald criteria 2017. Source: Thompson et al. (18).

Additional File 3 Relapse treatment of patients with MS. Source: Berkovich et al. (23, 26) and Correale et al. (21).

Additional File 4 Assessment of treatment response modified Rio score. Source: Sormani et al. (33, 34).
