# Supplementary figures and images for: Clinical Pathway for the Diagnosis and Management of Patients With Relapsing–Remitting Multiple Sclerosis: A First Proposal for the Peruvian Population
Source: Front Neurol. 2021 Oct 21;12:667398. doi: 10.3389/fneur.2021.667398 (PMC8567844; doi:10.3389/fneur.2021.667398)

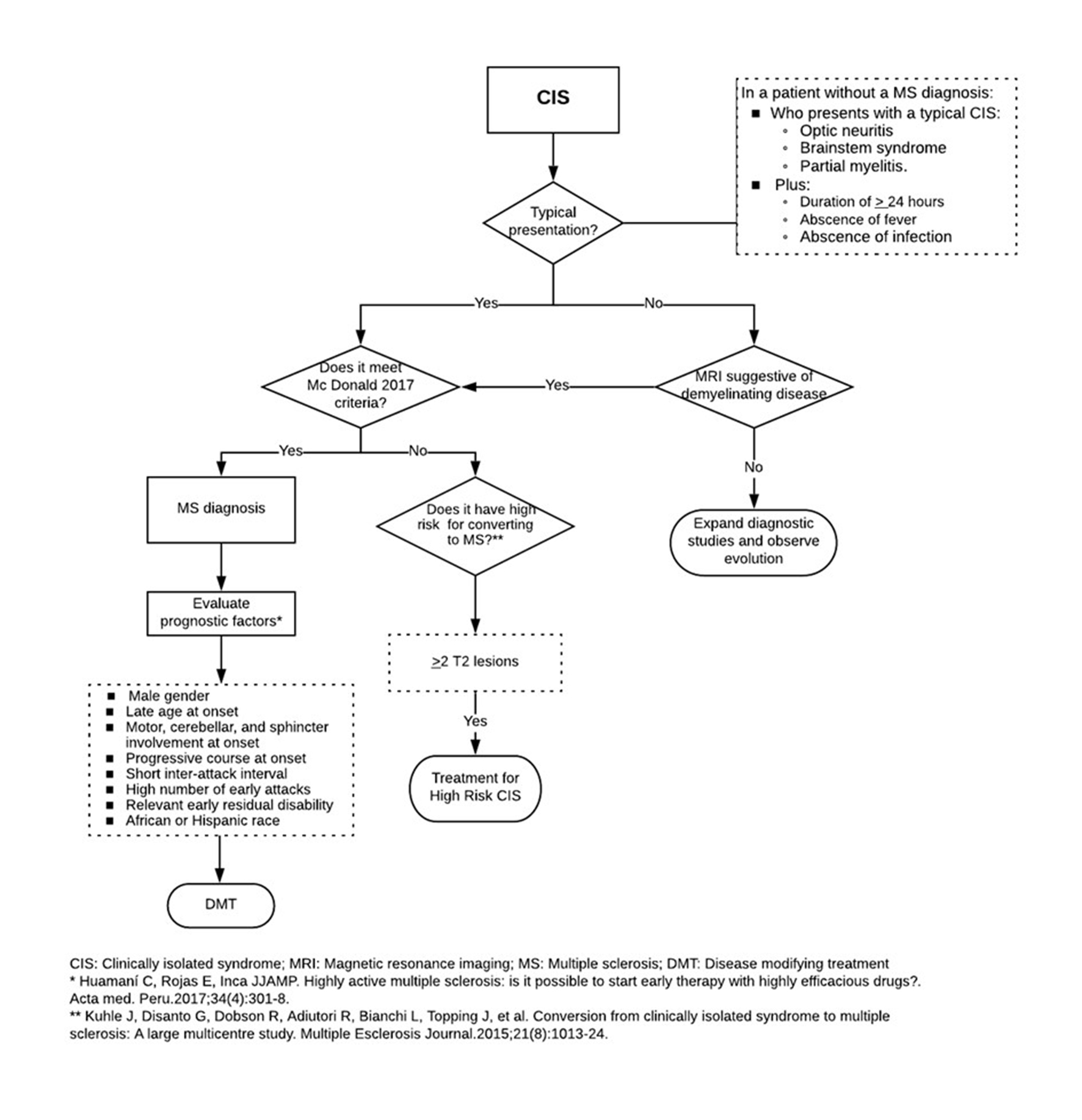

Supplement: Additional File 1 — MS diagnostic approach. Source: Huamani et al. (16) and Kuhler et al. (17). [file Image_1.JPEG]

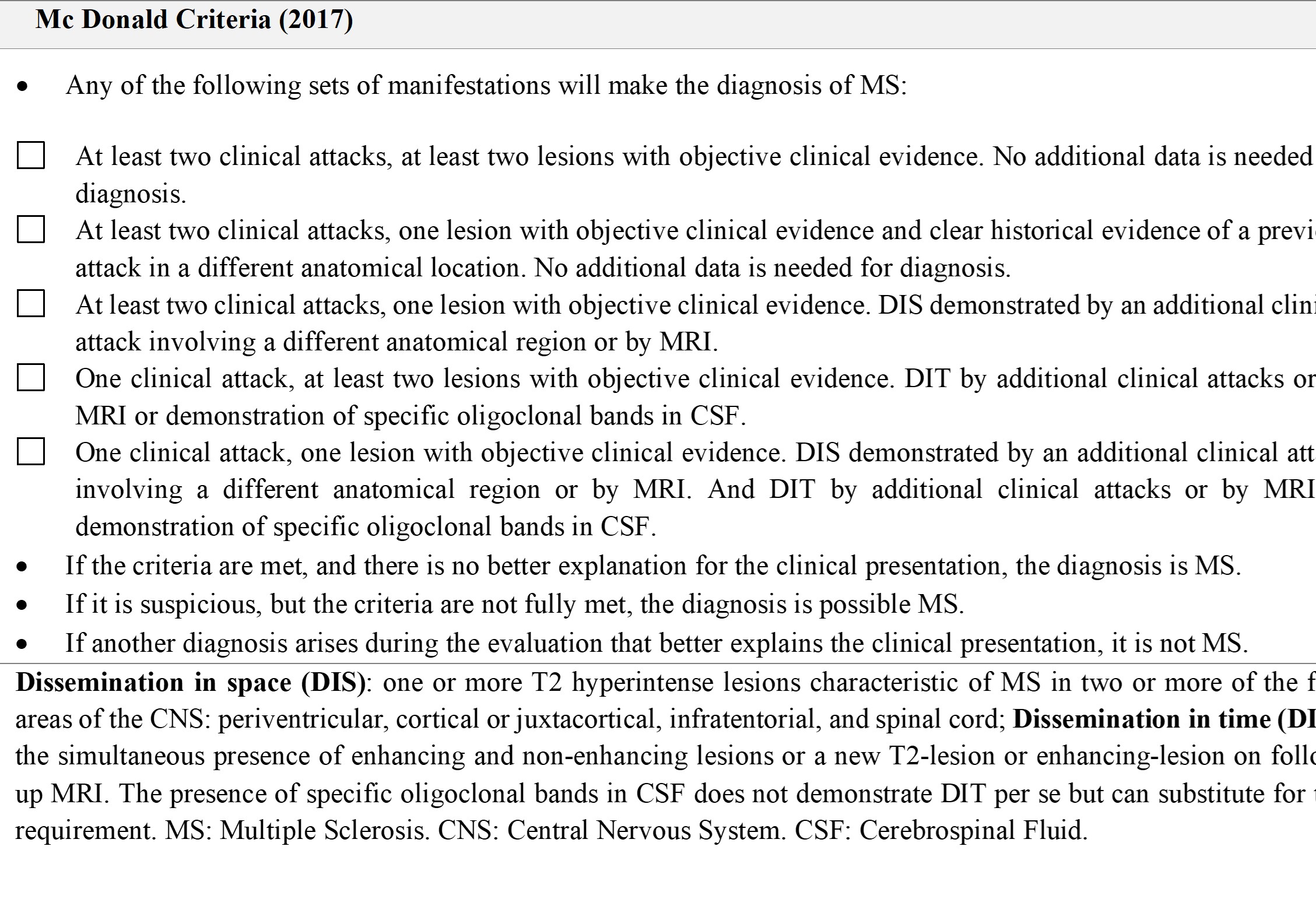

Supplement: Additional File 2 — Diagnostic protocol: Mc Donald criteria 2017. Source: Thompson et al. (18). [file Image_2.JPEG]

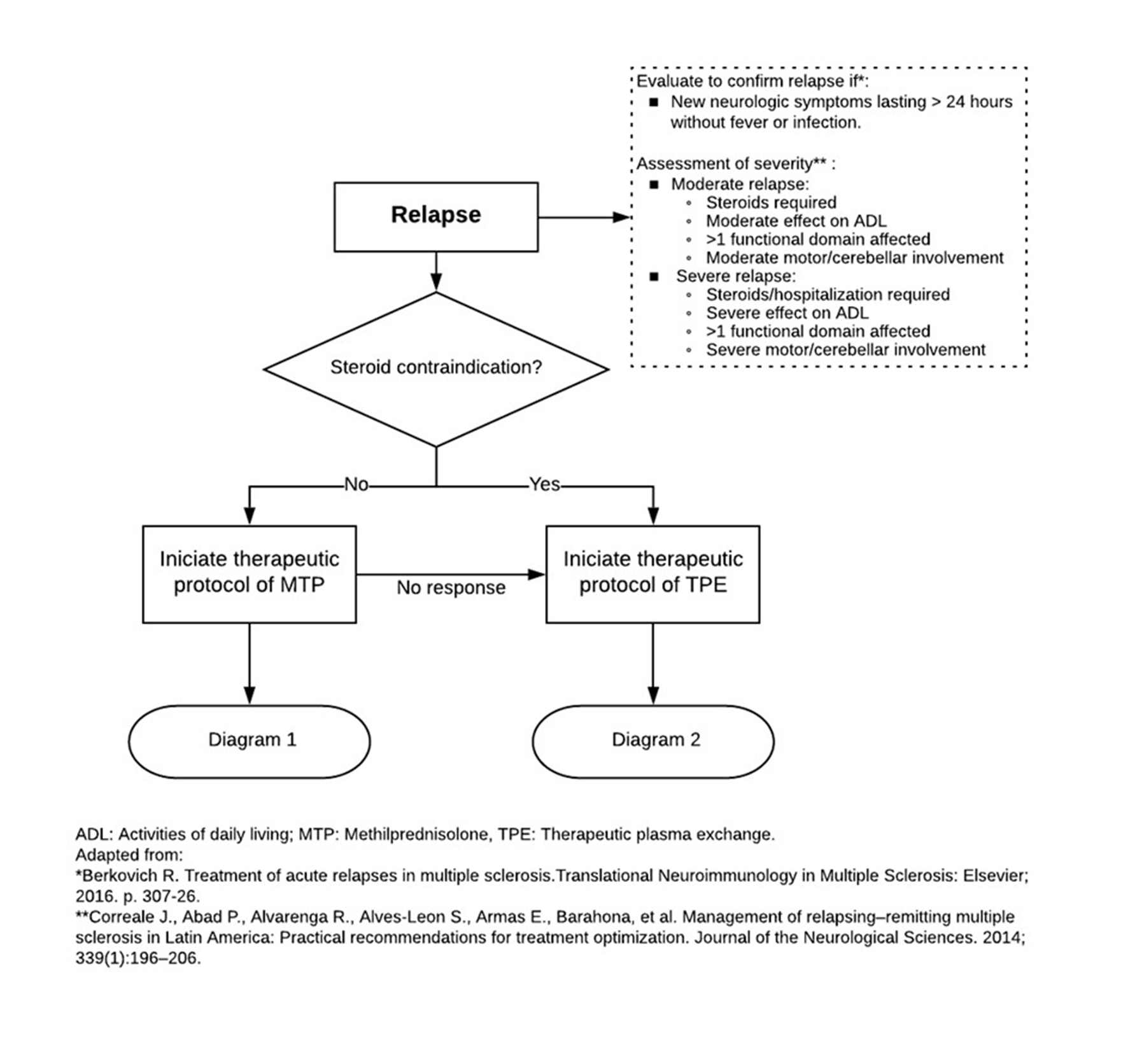

Supplement: Additional File 3 — Relapse treatment of patients with MS. Source: Berkovich et al. (23, 26) and Correale et al. (21). [file Image_3.JPEG]

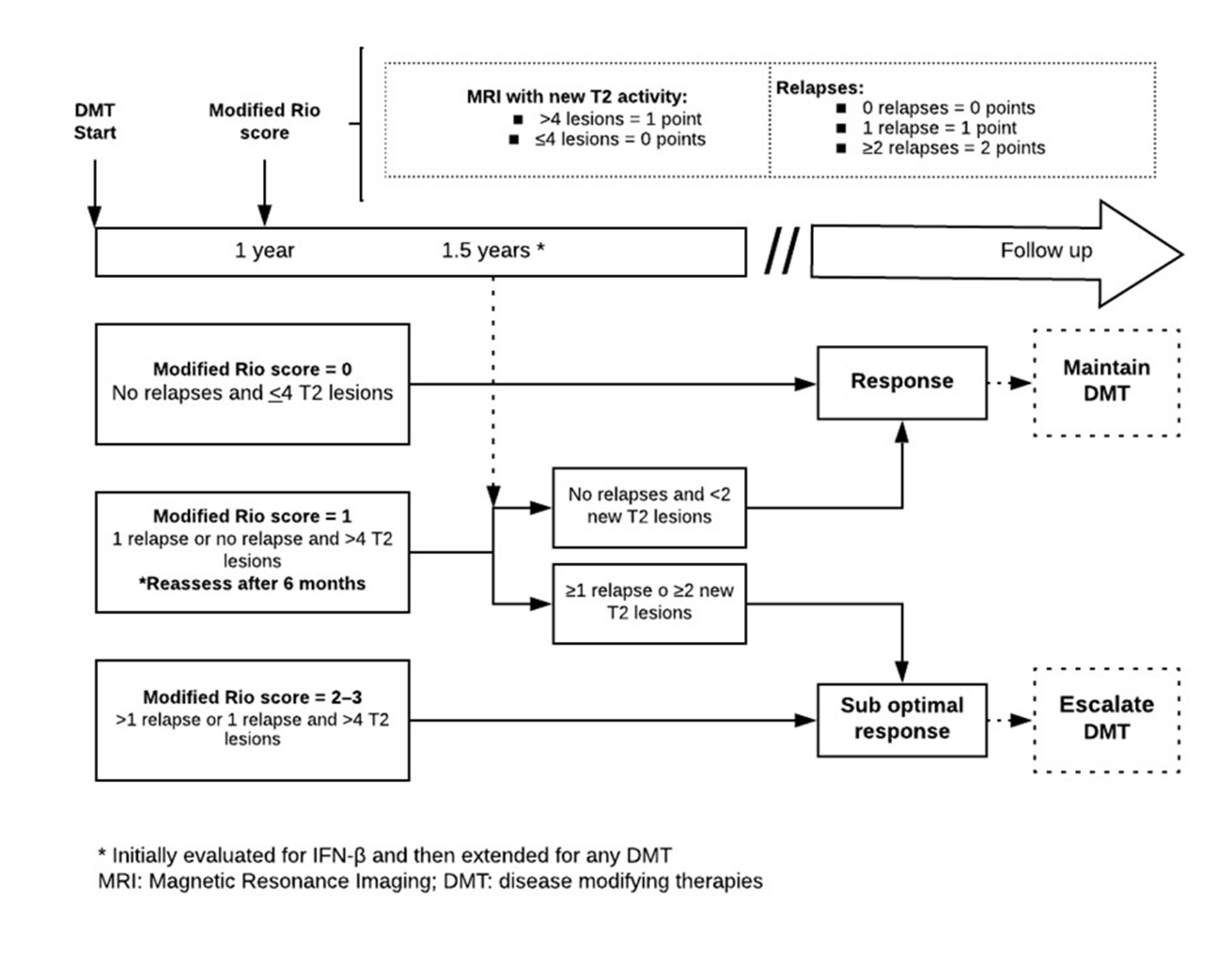

Supplement: Additional File 4 — Assessment of treatment response modified Rio score. Source: Sormani et al. (33, 34). [file Image_4.JPEG]
